# Smartphone-Based Whole-Cell Biosensor Platform Utilizing an Immobilization Approach on a Filter Membrane Disk for the Monitoring of Water Toxicants

**DOI:** 10.3390/s20195486

**Published:** 2020-09-25

**Authors:** Junning Ma, Dorin Harpaz, Yang Liu, Evgeni Eltzov

**Affiliations:** 1 Department of Postharvest Science, Institute of Postharvest and Food Sciences, the Volcani Center, Agricultural Research Organization, Bet Dagan 50250, Israel; junning.ma@hotmail.com (J.M.); dorin.harpaz@mail.huji.ac.il (D.H.); 2 Key Laboratory of Agro-Products Quality and Safety Control in Storage and Transport Process, Ministry of Agriculture and Rural Affairs, Institute of Food Science and Technology, Chinese Academy of Agricultural Sciences, Beijing 100193, China; liuyang@fosu.edu.cn; 3 Institute of Biochemistry, Food science and Nutrition, Faculty of Agriculture, Food and Environment, the Hebrew University of Jerusalem, Rehovot 76100, Israel; 4 School of Food Science and Engineering, Foshan University, Foshan 528231, China; 5 Agro-Nanotechnology Research Center, Agriculture Research Organization, the Volcani Center, Rishon LeZion 7505101, Israel

**Keywords:** bioluminescent bioreporter bacteria, whole-cell biosensor, smartphone signal detection, filter membrane immobilization, on-site detection, environmental water toxicants

## Abstract

Bioluminescent bacteria whole-cell biosensors (WCBs) have been widely used in a range of sensing applications in environmental monitoring and medical diagnostics. However, most of them use planktonic bacteria cells that require complicated signal measurement processes and therefore limit the portability of the biosensor device. In this study, a simple and low-cost immobilization method was examined. The bioluminescent bioreporter bacteria was absorbed on a filter membrane disk. Further optimization of the immobilization process was conducted by comparing different surface materials (polyester and parafilm) or by adding glucose and ampicillin. The filter membrane disks with immobilized bacteria cells were stored at −20 °C for three weeks without a compromise in the stability of its biosensing functionality for water toxicants monitoring. Also, the bacterial immobilized disks were integrated with smartphones-based signal detection. Then, they were exposed to water samples with ethanol, chloroform, and H_2_O_2_, as common toxicants. The sensitivity of the smartphone-based WCB for the detection of ethanol, chloroform, and H_2_O_2_ was 1% (*v*/*v*), 0.02% (*v*/*v*), and 0.0006% (*v*/*v*), respectively. To conclude, this bacterial immobilization approach demonstrated higher sensitivity, portability, and improved storability than the planktonic counterpart. The developed smartphone-based WCB establishes a model for future applications in the detection of environmental water toxicants.

## 1. Introduction

Over the past two decades, whole-cell biosensors (WCBs) have been widely used in the environmental, health-care, and agriculture sensing fields [1,2,3,4,5,6,7]. WCBs are constructed from three main parts: Bioreporter cells, interface (immobilization method), and transducer. The choice of the immobilization strategy is specific for each biosensor platform and mainly depends on the recognition element that is then linked to the transducer. Planktonic cells are the most common fixing approach that is used in WCBs biosensor platforms. However, these environmental toxicity monitoring assays that are conducted in the form of planktonic cells still present several drawbacks, including lower portability and storability, due to the requirement to regrow the bioreporter cells for each test. Other entrapment-encapsulation based immobilization approaches demonstrated higher activity of the immobilized cells as compared to the planktonic cells. Such examples include the immobilization of bacteria cells in gel-like matrices (e.g., sodium alginate and agar-agar) or on surfaces that enable the creation of a biofilm (e.g., optical fiber, gold, glass, and polystyrene) [8,9,10,11,12]. Bacteria cells that are immobilized in a biofilm state can display distinct physiological characteristics from that in the free-floating state [13]. For example, mature biofilm-based WCBs on polystyrene surfaces showed higher sensitivity in the presence of genotoxic substances than free-suspended bacteria [12]. Even though bacteria cells that are immobilized in hydrogels can maintain longer viability, surface immobilization can have better diffusion rates of analytes due to the use of a thinner membrane [11,14]. 

The immobilization of bacteria cells on surfaces is usually in a biofilm state, as a bacterial community [15]. The formation of bacterial biofilm consists of three main stages, namely initial adhesion, irreversible adhesion, and biofilm maturation [16]. Firstly, the bacterial adhesion occurs at the first 1–8 h after the bacteria cells come in contact with biotic or abiotic surfaces. The bacterial adhesion process is principally mediated by the net sum of nonspecific interactions, such as hydrophobic and electrostatic forces [15,17,18]. Subsequently, the maturation of the bacterial biofilm occurs after exopolysaccharides are produced, which tightly enclose the bacterial aggregates. The biofilm maturation process may take longer than 24 h to fully develop at certain conditions [12,17,18]. There are several factors that have an influence on the biofilm formation, such as temperature, humidity, surface properties, and additives [19,20,21]. Moreover, different surfaces possess different physical and chemical properties; thereby, leading to various binding affinities [20]. Also, additives such as sugars or plant extracts can affect bacterial adhesion efficiency by modulating the interaction between the bacteria and the surface [21,22,23]. Various surfaces (e.g., polystyrene and nylon mesh) have been examined and used for the immobilization of bacterial cells in WCBs for environmental monitoring or bioremediation applications [12,24,25]. A common surface material is the cellulose-based filter membrane that is very cheap and easily accessible as a lab material and it is used in a wide range of fields. A cellulose-based filter membrane disk was used for the long-term preservation of bacteria cells for up to four years, as well as for the preservation for up to one year of DNA from humans, bacteria, and insects [26,27,28]. Nonetheless, the immobilization of bioluminescent bioreporter bacteria by utilizing a filer membrane disk was not yet reported. 

In this study, a simple and low-cost bacterial immobilization strategy was developed. The bacteria cells were immobilized by adsorption on a filter membrane disk. Then, the bioluminescent bacterial functionality was tested for the ability to monitor water toxicity. The integration of a WCB on a filter membrane disk with portable signal detection can further expand the access and use of the sensing platform in low-resource regions. Smartphones have been increasingly used as an attractive alternative to expensive and bulky signal detection equipment for luminescence or fluorescence measurement. Among the smartphone advantages are its portability, cost-effectiveness, high performance of CMOS sensor, CPU, GPU, and excellent internet-connectivity [29]. Smartphone-based biosensors have been extensively applied in the fields of environmental monitoring [30,31,32], point-of-care diagnostics [33,34], and food safety [35,36]. Even so, due to the low light signal that is produced by the bioluminescent bacteria, the integration of a smartphone-based bioluminescence signal measurement in WCBs requires customized setup [35,37]. Therefore, in this study, the feasibility of integrating smartphone-based signal detection with WCB that utilizes a filter membrane disk with immobilized bioluminescent bacteria was examined for the monitoring of water toxicity. The integrated, portable, and low-cost WCB device could enable improved sensing potential with a wide application range in the field of environmental monitoring.

## 2. Material and Methods

### 2.1. Materials

Absolute ethanol was purchased from Gadot-Group (Netanya, Israel). Difco^TM^ LB powder was used for the preparation of a working concentration of LB broth (20 g/L) and was purchased from Becton, Dickinson, and Company (Franklin Lakes, NJ, USA). Ampicillin sodium salt was purchased from Fisher Scientific (Waltham, MA, USA). D-Glucose anhydrous was purchased from Fisher Chemical (Waltham, MA, USA). Chloroform was purchased from Bio-Lab (Jerusalem, Israel). H_2_O_2_ was purchased from Carlo Erba Reagents (Barcelona, Spain). Whatman^®^ Grade 1 qualitative filter paper was purchased from Sigma-Aldrich (St. Louis, MI, USA). Parafilm was purchased from Bemis Company (Neenah, WI, USA). Polyester cloth (PT-R5) was purchased from MDI Membrane Technologies (Ambala Cantt, India).

### 2.2. Bacterial Strains and Growth Conditions

Four genetically engineered bioluminescent bacterial strains from *E. coli* were examined: DPD2794, TV1061, *grpE::lux,* and *soxS::lux*, which were received from Prof. Shimshon Belkin (Hebrew University, Jerusalem, Israel) [38,39,40,41]. Firstly, they were grown overnight in 5 mL Difco^TM^ LB broth (20 g/L) supplemented with 100 μg/mL ampicillin on a shaker (Lumitron, Petah Tikva, Israel) at 120 rpm and 37 °C. A 100 μL aliquot (1/100 dilution) of the overnight-grown bacteria was subcultured in 10 mL LB broth on a shaker without shaking at 37 °C for 3 h. Then, the bacterial culture was centrifuged at 5000 rpm for 10 min, to achieve an optical bacteria cell density of 1.2 at 600 nm (OD_600_). It was then diluted to the desired concentration of 1.0, 0.8, 0.6, 0.4, and 0.2, as determined in an Ultrospec 2100 pro spectrometer (Amersham, UK).

### 2.3. Surface Adsorption of Bacteria Cells and Signal Measurement Procedure

The immobilization of the bioreporter bioluminescent bacteria cells was examined on several different surfaces. The filter membrane disks in a diameter of 0.5 mm were created by punching into parafilm and polyester membranes. Then, a 50 μL bacterial suspension with or without additives (100 μg/mL ampicillin and 10 mM glucose) at different concentrations was immobilized onto the filter membrane surface disks by incubation in petri dishes in an incubator at 37 °C for 2 h, until the solution fully evaporated. The filter membrane disks with the immobilized bacteria were subsequently ready for the monitoring of toxicants in liquid or long-term storage at room temperature. The bacterial bioluminescence activity was measured in a Synergy^HTX^ multi-mode reader (BioTek Instruments Inc., Winooski, VT, USA), by placing it in white opaque 96-well microtiter plates (Nunc, Roskilde, Denmark). The plates containing the bacteria immobilized surface disks were exposed to different concentrations of toxicants in LB and control samples (*n* ≥ 3 for each tested condition). During the measurement, the temperature of the samples was maintained at 26 °C. The luminescence values are presented in relative light units (RLU).

### 2.4. CMOS-Based Bioluminescence Measurement and Data Analysis

The filter membrane disks were integrated with a CMOS sensor (Anitoa 4-Channel ULS24 Solution Kit, Menlo Park, CA, USA) to develop a remote and on-site detection of water toxicity [8]. Firstly, the disks were immobilized with the selected bacterial strain TV1061. Then, LB solution (as a control group) and 2% (*v*/*v*) ethanol dissolved in LB (as analyte sample) were also added to the filter membrane disks. Thereafter, the kinetic measurement of the bioluminescence signal started after 2 h of incubation. The data processing was conducted by using python (version 3.7.3) script (Supplementary Material). The data analysis included the following steps: (1) Extraction of temperature reading for each measurement; (2) data extraction for each channel; (3) calculation of background signal reading according to the standard curve [8]; (4) calibration of the bacterial signal by the subtraction of the calculated background signal; (5) computation of the induction factor; (6) and reconstruction of the output images.

### 2.5. Smartphone-Based Bioluminescence Measurement and Data Analysis

**Figure 1:**
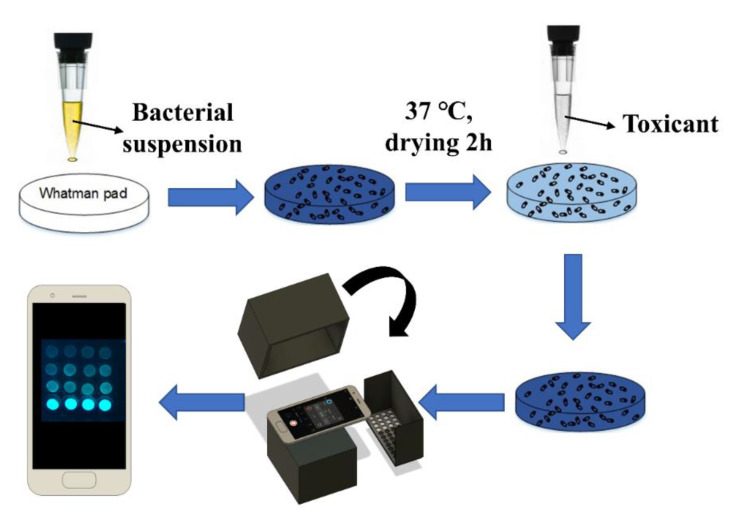
Bioluminescence signal measurement procedure of smartphone-based whole-cell biosensor.

**Figure 2:**
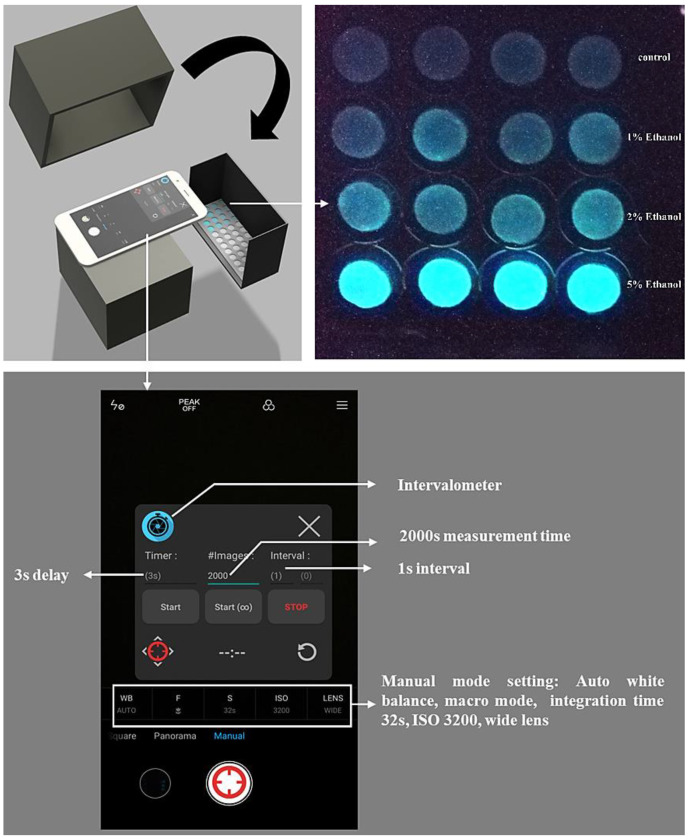
Detailed setup of the smartphone-based whole-cell biosensor.

**Figure 3:**
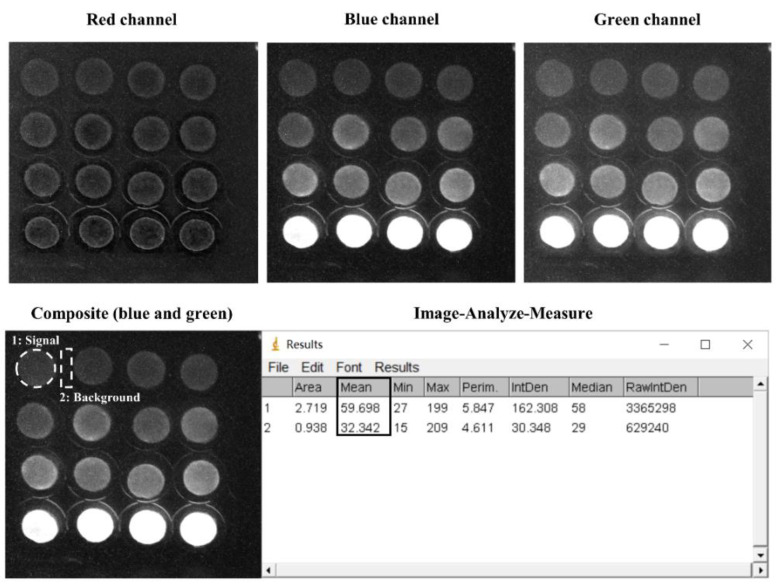
Exemplary data analysis of smartphone-taken images of the immobilized bioreporter bacteria on the filter membrane disks.

The smartphone-based measurement procedure is presented in Figure 1, and the setup is presented in Figure 2. The android smartphone Mi 6 (Xiaomi, Beijing, China) was used for the bioluminescence measurement with the camera setting: Manual mode, auto white balance, macro mode (a focal distance of approximately 75 mm), the integration time of 32 s, ISO 3200, and wide lens (Figure 2). Also, the commercial application Intervalometer (version 2.66, MobilePhoton) was purchased from google play store with the following setting: 3 s of shutter delay, 2000 s of measuring time, and frame interval of 1 s. Firstly, the surface disks with the adsorbed bacteria were arranged in a customized black box that was folded from a carton paper and then pasted with a black ink-printed paper (Figure 2). Then, 50 μL of different concentrations of each toxicant were diluted in LB solution and placed on the filter membrane disks. Following this, 2000 s of smartphone measurement were conducted, they were centered by the peak time as previously determined by the measurement in the plate-reader. The measurement process was conducted in a light-tight box to minimize the interference from environmental light signals. Moreover, to maximize the bioluminescence bacterial signal, 60 images that were taken by the smartphone were then stacked into one image by computing the average of all 60 images using the python script (Supplementary Material). To further increase the signal-noise ratio, green and blue channels (Image-Color-Split Channels) from the stacked image were merged to calculate the mean integrated intensity in ImageJ (Figure 3). Also, the background signal was manually selected and subtracted from the bacterial light signal.

### 2.6. Statistics Analysis

The bioluminescence bacterial signal indicates the response of the bacteria to the different tested parameters. The signal was expressed as an induction factor, which was calculated as Bi/Bc, where Bi is the maximum bioluminescence signal of the tested toxicant and Bc is the maximum signal of the control. The signal-noise ratio was calculated as the average induction factor divided by the standard deviation from at least three replicates. For each experiment, a minimum of three separate replicates were conducted to ensure the system reproducibility. A paired *t*-test was employed to compare the means between two groups and a one-way ANOVA with Fisher’s Least Significant Difference (LSD) was used for the comparison of means between three or more groups. The error bars represent the standard error of three independent replicates and the different letters in the figures indicate a different level of statistically significant difference (“*” when *p* < 0.05 and “**” when *p* < 0.01). 

## 3. Results and Discussion

### 3.1. Higher Sensitivity and Stability of Immobilized Bacteria Cells on Filter Membrane Disks

**Figure 4:**
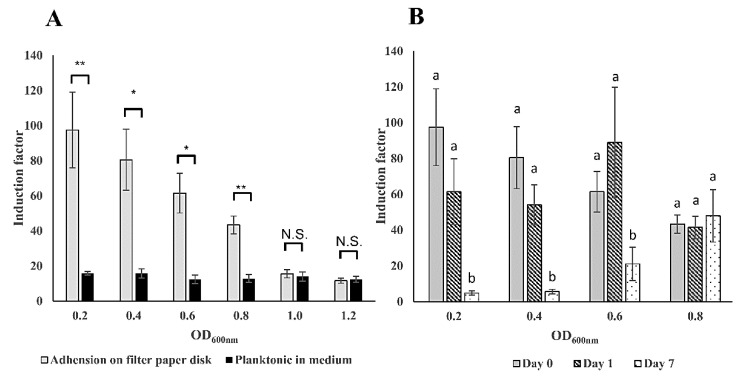
Sensitivity and stability of the immobilized bacteria cells on filter membrane disks. (**A**) Comparison of the sensitivity to 2% (*v*/*v*) ethanol between filter membrane adhered and planktonic forms of *E. coli* luminescent strain TV1061 in different optical densities at OD_600 nm_. (**B**) Stability of the sensitivity to 2% (*v*/*v*) ethanol during storage of filter membrane adhered *E. coli* strain TV1061 at −20 °C for one day and seven days. All the values in the graphs are presented as mean ± standard error of the mean (SEM), with “*” for *p* < 0.05, “**” for *p* < 0.01, and “N.S.” for “Not Significant” (*p* > 0.05), which were calculated by using the student’s *t*-test. Different letters above the bars indicate significant differences (*p* < 0.05) that were tested by the Fisher’s Least Significant Difference.

Bacterial cells in the planktonic state show different physiology from that in the immobilized state on surfaces [42,43]. This may also result in a different sensitivity of the bioreporter bacteria to toxicants. In order to examine the immobilization matrices, a comparison of the sensitivity to 2% (*v*/*v*) ethanol between filter membrane adhered and planktonic forms of *E. coli* luminescent strain TV1061 in different optical densities at OD_600 nm_ was conducted (Figure 4). The results demonstrate that the sensitivity to the model cytotoxicant ethanol was 6.1, 5.1, 4.9, and 3.4 times higher at OD_600_ of 0.2, 0.4, 0.6, and 0.8, respectively, in the case of the immobilized bioluminescent bacterial strain TV1061 on filter membrane disk by adsorption, in comparison with that in free-floating bioluminescent bacteria (Figure 4A). These findings are supported by previously reported results that showed evidence for enhanced sensitivity in surface-bound *E. coli* strain AB725 as compared to suspended ones when tested with genotoxicant nalidixic acid [12]. The explanation behind this higher sensitivity might be the cooperativity between the immobilized bacteria cells, which maximizes the bioluminescence signal output when exposed to environmental toxicants [15,44,45]. It was previously reported that the transition of bacterial cells from a free-swimming state to adsorption on surfaces might lead to changes in their cellular responses as well as to cooperative behavior between the bacteria cells, due to the limitation of nutrients [44]. From the results, the highest sensitivity was observed at the bacteria cells concentration at an optical density of 0.2 (OD_600_). Nonetheless, the preferred immobilization strategy should maintain a stable sensitivity for an extended storage period. Therefore, the stability of the sensitivity to 2% (*v*/*v*) ethanol during storage was compared at −20 °C for one day and seven days (Figure 4B). The results show that lower bacterial density (e.g., OD_600_ = 0.2, 0.4, and 0.6) resulted in significantly lower sensitivity by 65–95% within one-week of storage period. Moreover, a higher density of bacteria (OD_600_ = 0.8) demonstrated a more stable sensitivity to ethanol during storage. It is possible that the interactions between the bacteria cells in higher density might compensate for the loss of bacterial viability that results from the lack of water and nutrient during the storage period. More importantly, the higher density of bacteria may enhance the interaction between the cells and thereby maintain the stability of the bacterial population [44,46]. To conclude, the immobilization of the bioreporter bacteria at an optical density of 0.8 (OD_600_) on the filter membrane disk demonstrated higher sensitivity to ethanol and also maintained better stability during storage; therefore, it was chosen as the preferred bacterial density for subsequent analysis. 

### 3.2. Filter Membrane Disks as the Preferred Surface Material for Bacterial Immobilization

**Figure 5:**
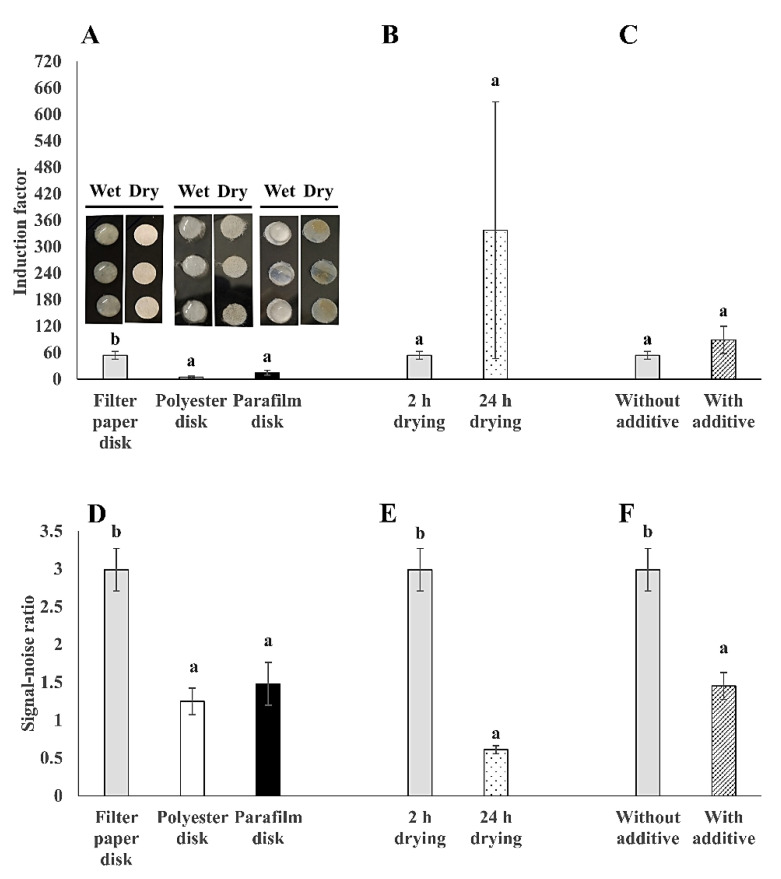
Comparison between different membrane disks as immobilization matrices. Induction factor values of (**A**) three surface materials: Filter membrane, polyester membrane, and parafilm disks; (**B**) effect of drying time on filter membrane disks; (**C**) effect of additives: 10 mM glucose and 100 μg/mL ampicillin on filter membrane disks, on the sensitivity of surface-immobilized *E. coli* bioreporter strain TV1061 to 2% (*v*/*v*) ethanol; and signal-noise ratio values of (**D**) three surface materials; (**E**) effect of drying time on filter membrane disks; (**F**) effect of additives on filter membrane disks on the sensitivity of immobilized TV1061 to 2% (*v*/*v*) ethanol. All the values in the graphs are presented as mean ± standard error of the mean (SEM), with different letters above the bars that indicate significant differences (*p* < 0.05), which were tested by the student’s *t*-test (**B**,**C**,**E**,**F**) and the Fisher’s Least Significant Difference (**A**,**D**).

In order to develop a simple, cheap, and stable strategy for the immobilization of the bioreporter bacteria cells, commonly used lab materials (e.g., filter membrane disks) were examined as immobilization matrices. Different surface materials have distinct physical and chemical properties; therefore, they have different effects on the immobilization of bacteria cells onto surfaces [20]. In this study, three different surface materials, filter membrane, polyester membrane, and parafilm, were compared for their use as immobilization matrices for the tested bioreporter bacteria (Figure 5). Both induction factor and signal-noise ratio values were compared for the tested parameters. The results show that the bioluminescent bacteria that was adsorbed on the filter membrane disks demonstrated the highest sensitivity to 2% (*v*/*v*) ethanol, with 4 to 11 times higher sensitivity than the other two surface materials (Figure 5A). Also, the signal–noise ratio was doubled in filter membrane immobilized bacteria than in the polyester and parafilm counterparts (Figure 5D). This might be attributed to the stronger binding interaction of the bacteria cells to the cellulose filter membrane than to the other two surface materials. The surface binding affinity is dependent on the net sum of various forces, such as hydrophobic, electrostatic, and Van der Waals force [15,17,18]. It was previously reported that surface–bacteria interactions are predominantly determined by hydrophobic forces [47]. The filter membrane disk surface is hydrophilic, while the surfaces of parafilm and polyester membrane disks are hydrophobic. Most importantly, evidence was found to support the hydrophilic nature of *E. coli* because it is dominated by extracellular polysaccharides that have a water contact angle below 40° [48]. Therefore, the explanation behind the differences in the signal-noise ratio values between the three tested surface materials might be due to the interactions between the immobilized hydrophilic *E. coli* bacteria with the hydrophilic filter membrane surface. Hence, the filter membrane disks were found to be preferred for bacterial immobilization. Moreover, the drying process can also facilitate bacterial attachment to the surface by the evaporation of water molecules [49]. Previous studies examined various incubation times from 1 to 48 h in order to allow bacterial surface attachment [12,17,18]. Therefore, the drying time of 2 vs. 24 h was also examined for the different tested bacterial strains (Figure 5B,E). From the results, it was observed that longer drying time (24 h) on filter membrane disks resulted in similar sensitivity (Figure 5B), but with 5 times lower signal-noise ratio (Figure 5E) than that of 2 h drying. This can be explained by the possible bacterial cell death in the case of a long drying period, especially in the membrane upper layers, which then results in a light signal gradient through the biofilm layers. As previously mentioned, bacterial attachment process consists of three main stages, namely initial attachment, irreversible attachment, and biofilm maturation, which develop progressively with the incubation time [16]. Additional parameters, such as the physiological state of the bacteria cells, membrane thickness, oxygen, and nutrient gradient, may also contribute to the differences between the light signals that were received in the 2 and 24 h dried bacterial membranes disks [18,50,51,52]. Therefore, a 2 h incubation and drying process at 37 °C was found to be sufficient for the bacterial adhesion to the filter membrane disks, producing a stable light signal. Moreover, the addition of additives such as sugars can modulate the physico-chemical properties of surfaces, which may influence on the bacterial binding and biofilm formation; this effect is largely dependent on the bacteria type [21]. Therefore, the effect of two additives (100 μg/mL ampicillin and 10 mM glucose) was also examined. From the results, a similar sensitivity to 2% (*v*/*v*) ethanol was received in the additives-supplemented bacteria and in the non-supplemented bacteria (Figure 5C). Most importantly, the additives-supplemented bacteria showed half of the signal-noise ratio than in the case of bacteria without additives (Figure 5F). A possible explanation to this effect might be due to the growth-promoting effect of glucose on the bacteria. Even though the glucose addition in the medium can promote *E. coli* biofilm formation on surfaces at certain conditions, the oxygen homogeneity in the membrane might be another limiting factor that is affecting the bioreporter signal stability [43,53]. To conclude, the bacteria cells were immobilized onto the filter membrane disks without the addition of glucose and ampicillin, as the preferred approach for further testing.

### 3.3. Effect of Storage Temperature and Duration on the Bacteria Sensitivity

**Figure 6:**
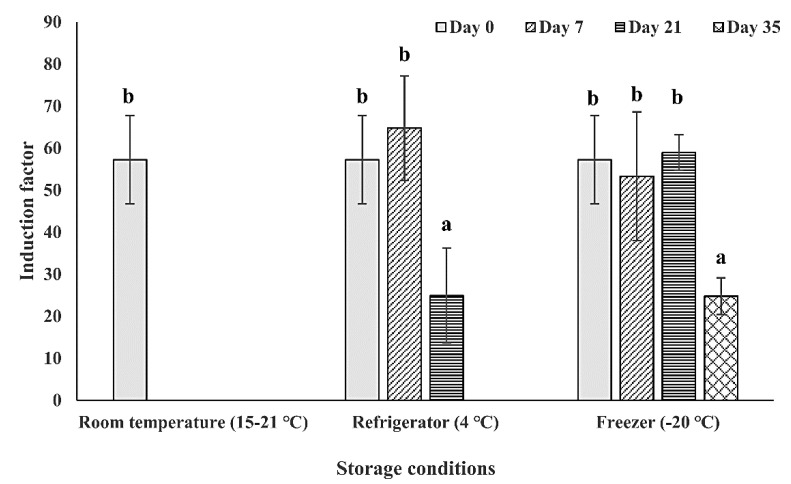
Effect of the storage temperature and duration on the bacterial sensitivity. The bioreporter bacteria cells from strain TV1061 were immobilized on the filter membrane and stored in different temperatures: Room temperature (15–21 °C), 4 °C, and −20 °C for the different durations (0, 7, 21, and 35 days). Their sensitivity to 2% (*v*/*v*) ethanol was then tested. All the values in the graph are presented as mean ± standard error of the mean (SEM), with different letters above the bars that indicate significant differences (*p* < 0.05), which were tested by the Fisher’s Least Significant Difference.

After the determination of the immobilization conditions of the bioreporter bacteria on the filter membrane disk surface, the storage temperature (room temperature (15–21 °C), 4 °C, and −20 °C) and duration (0, 7, 21, and 35 days) was further examined (Figure 6). From the results, the tested immobilized bioreporter bacteria on filter membrane disks were more stable when it was stored in lower temperatures (4 °C and −20 °C). While the storage of the bacteria at room temperature resulted in sensitivity stability only on the first time point of day 0, the bacteria storage at 4 °C allowed the bacteria to be stable for another week without a decrease in its sensitivity level. Also, the storage at −20 °C increased the bacteria stability for another week, maintaining its sensitivity also in the 21-day time point, and with its sensitivity decreasing by half only in the 35-day time point. The results received are as expected because the lower temperature can slow the rate of the metabolic activity of the bacteria; thereby, slowing down the speed of nutrient and water depletion in the bacterial biofilm. Another possible explanation is that the low temperature of storage can maintain the biofilm attachment on the surface, while high temperature facilitates the biofilm dispersion [54,55]. To conclude, the surface-attached bacteria could maintain its highest detected biosensing stability under the storage conditions of −20 °C within three weeks and decreasing to half of its sensitivity after five weeks of storage.

### 3.4. Whole-Cell Biosensor Performance for the Detection of Toxicants in Water Samples

**Figure 7:**
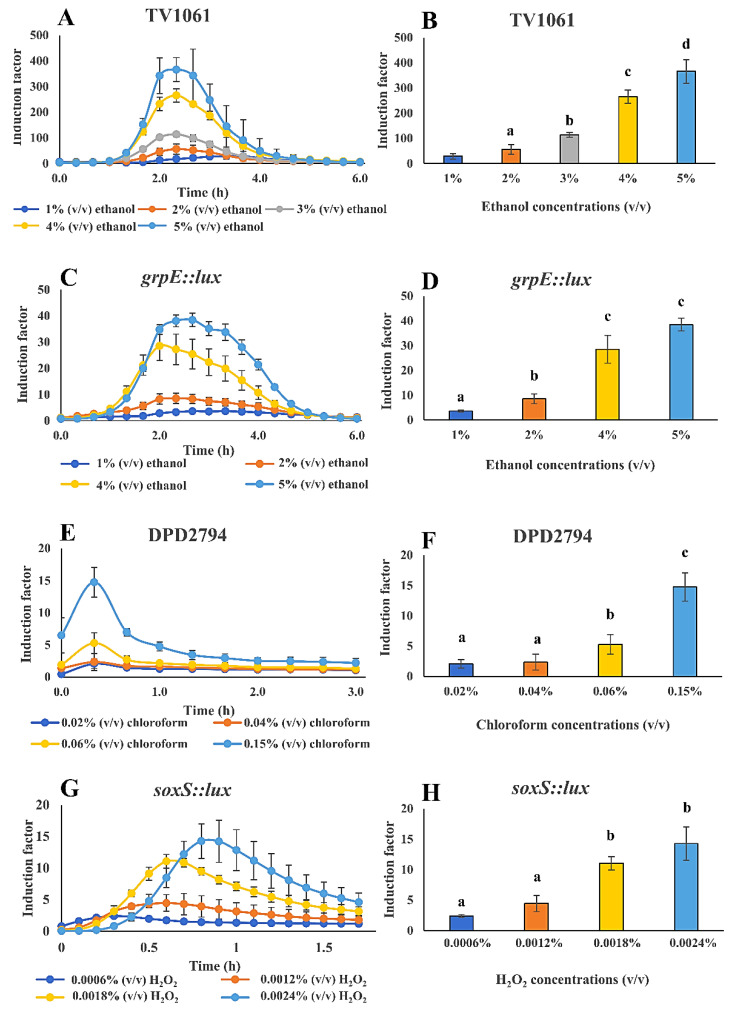
Whole-cell biosensor performance for the detection of toxicants in water samples using microplate reader for light signal detection. Detection of the following toxicants in spiked water samples: (**A**–**D**) Ethanol with TV1061 and *grpE::lux*; (**E**,**F**) chloroform with DPD2794; (**G**,**H**) hydrogen peroxide (H_2_O_2_) with *soxS::lux*. All the values in the graphs are presented as mean ± standard error of the mean (SEM), with different letters above the bars that indicate significant differences (*p* < 0.05), which were tested by the Fisher’s Least Significant Difference.

The WCB was specifically characterized for the immobilization on filter membrane disks and storage. Thereafter, to further evaluate the WCB performance in the detection of toxicants in water samples, a luminescence microplate reader was used for the light signal measurement after the WCB exposure to different concentrations of toxicants in water samples (Figure 7). Also, the peak time of the light signal was examined and determined, for it to be used later for the CMOS-based and smartphone-based light signal measurement. Four bioreporter bacterial strains were applied, including TV1061 that is sensitive to cytotoxicants; *grpE:lux* that is also sensitive to cytotoxicants; DPD2794 that is sensitive to genotoxicants; and *soxS::lux* that is sensitive to oxidants. In the results, visible dose-dependent patterns are shown for the detection of all the tested toxicants in spiked water samples: Ethanol, chloroform, and H_2_O_2_. In detail, the immobilized bacterial strain TV1061 on the filter membrane disks presented a sensitivity 30, 56, 114, 265, and 366 times higher to 1%, 2%, 3%, 4%, and 5% (*v*/*v*) of ethanol, respectively, as compared to the control sample, demonstrating a peak signal at 2.5 h after incubation (Figure 7A,B). In the case of immobilized bacterial strain *grpE::lux*, its sensitivity to 1%, 2%, 3%, and 5% (*v*/*v*) of ethanol was 3.7, 8.5, 28.5, and 38.5 times higher, respectively, than the control sample, with a peak signal at 2.3 h (Figure 7C,D). Likewise, the exposure of the immobilized bacterial strain DPD2794 on the filter membrane disks to 0.02%, 0.04%, 0.06%, and 0.15% (*v*/*v*) of chloroform resulted in induction values 2.1, 2.3, 5.3, and 14.8 times higher, respectively, as compared to the control sample, with a peak signal at 0.3 h after incubation (Figure 7E,F). Also, for the case of immobilized bacterial strain *soxS::lux*, its response to 0.0006%, 0.0012%, 0.0018%, and 0.02% (*v*/*v*) of H_2_O_2_ was 2.4, 4.5, 11.1, and 14.3 times higher, respectively, as compared to the control, with a peak signal between 0.3 h and 0.8 h (Figure 7G,H). The results validate the feasibility of the WCB that is immobilized on the filter membrane disks for the detection of a variety of toxicants. It also lays the foundations for the later integration with portable light signals measurement transducers, such as CMOS and smartphone. The determined peak signal time points for each bacterial strain will be later used to determine the start and end time point (peak time point ±1000 s, except for strain *soxS::lux*) in the process of the smartphone-based light signal measurement. Specifically, for the bacterial strain TV1061, the measurement time interval for ethanol detection was from approximately 2.2 h to 2.7 h, while the time interval for the monitoring of ethanol using the immobilized bacterial strain *grpE:lux* was from 2 h to 2.5 h after the exposure to the toxicant. Also, for the case of the bacterial strain DPD2794, the start and end time points for chloroform detection was from 1 min to 31 min. Specifically, the time for monitoring H_2_O_2_ using the bacterial strain *soxS::lux* was from 0.3 h to 0.8 h after incubation. To conclude, these findings determine the time interval that is used for downstream smartphone-based monitoring of toxicants in water samples, as well as demonstrates the wide applicability of the filter membrane immobilized WCBs in environmental monitoring. 

### 3.5. Comparison Between CMOS-Based and Smartphone-Based Light Signal Measurement

**Figure 8:**
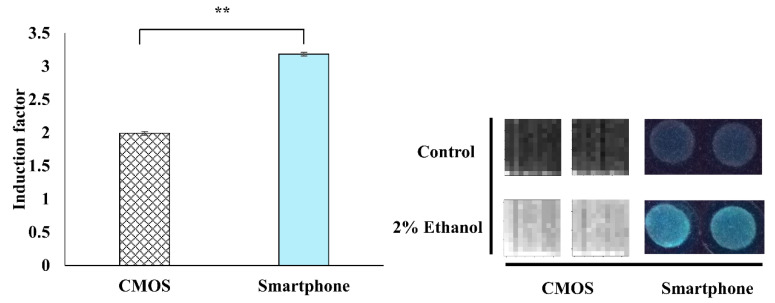
Comparison between CMOS-based and smartphone-based luminescent light signal detection. The response of the bioreporter bacterial strain TV1061 was examined in exposure to 2% (*v*/*v*) ethanol. All the values in the graph are presented as mean ± standard error of the mean (SEM), with “**” for *p* < 0.01 that was tested by the student’s *t*-test.

The developed WCB was integrated with low-cost and portable transducers for the light signal measurement. Two transducers were examined and compared, CMOS and smartphone (Figure 8). The CMOS light signal measurement approach was used with a ULS24 solution kit with a 4-channel ultra-low-light CMOS image sensor. The CMOS approach presents the limitation of low signal-noise ratio in terms of bacterial bioluminescence measurement [8]. The second tested approach was the use of a smartphone that is more commonly available, together with high-performance CMOS sensors and assembly of optical lens. In this study, the WCB was integrated with a lensless CMOS detector and a smartphone (Figure 2). From the results, it is possible to determine that the CMOS-based sensor measured the produced bioluminescence signal (in the presence of 2% (*v*/*v*) ethanol as the model toxicant) with approximately 1.6 times lower sensitivity as compared to the Mi 6 smartphone (Figure 8). The induction factor reported by the CMOS-based sensor was around 2, while the induction factor reported by the smartphone-based sensor was around 3.2. Another advantage of the smartphone-based over the CMOS-based sensor is that the images that are produced from the smartphone are three-channel RGB images, which enables the removal of red channel (irrelevant light signal to emitted bacterial blue-green light signal). Meanwhile, the CMOS sensor captures only one-channel grayscale images, which gives the total light intensity. This important advantage might contribute to the higher sensitivity that was received in the smartphone-based sensor. Another possible explanation might be the presence of a lens in the smartphone, which facilitates an optimal focus on the target and, therefore, more sensitive detection of the light signal. Lensless CMOS sensors can also function accurately, but this requires proper focal distance to the object and implementation of the reconstruction algorithm [56]. To conclude, the smartphone-based measurement of the bioreporter bacterial bioluminescence signal is the preferred option to be integrated into a portable monitoring device.

### 3.6. Whole-Cell Biosensor with Smartphone-Based Setup Performance for the Detection of Toxicants in Water Samples

**Figure 9:**
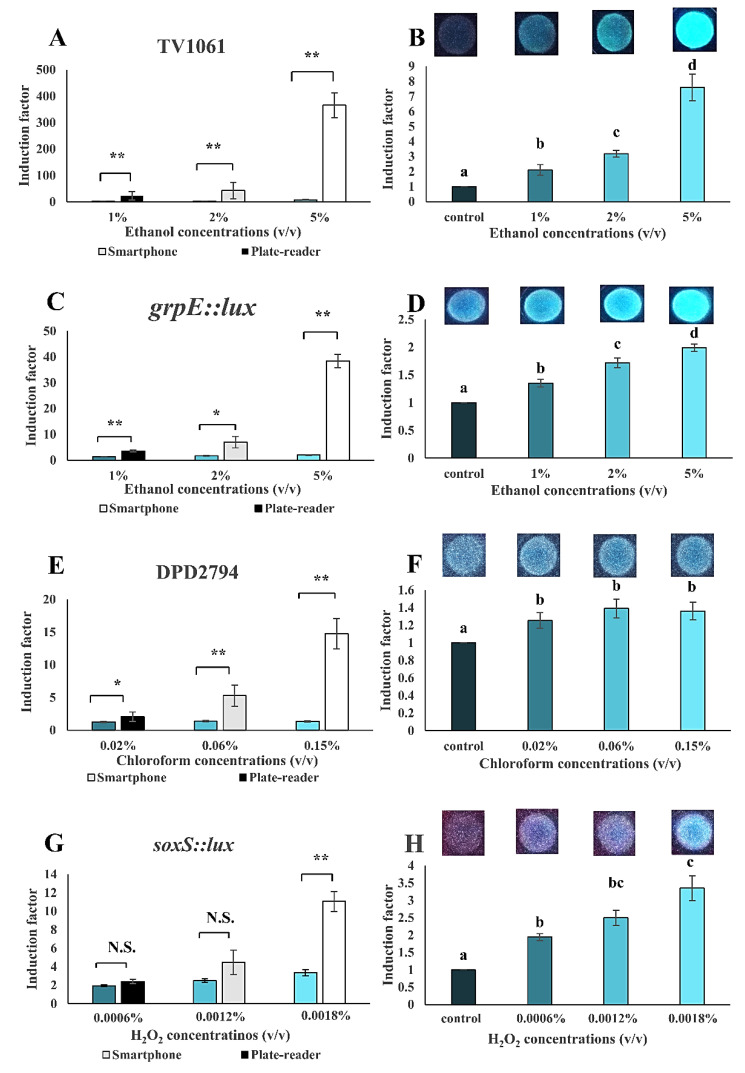
Smartphone-based setup performance in the detection of toxicants in water samples. Detection of the following toxicants in spiked water samples: (**A**–**D**) Ethanol with TV1061 and *grpE::lux*; (**E**,**F**) chloroform with DPD2794; (**G**,**H**) hydrogen peroxide (H_2_O_2_) with *soxS::lux*. All the values in the graphs are presented as mean ± standard error of the mean (SEM), with “*” for *p* < 0.05, “**” for *p* < 0.01, and “N.S.” for “Not Significant” (*p* > 0.05), which were calculated by using the student’s *t*-test. Different letters above the bars indicate significant differences (*p* < 0.05) that were tested by the Fisher’s Least Significant Difference.

The WCB was assembled into a portable setup with smartphone-based signal measurement. The proof-of-concept was done for the detection of ethanol, chloroform, and H_2_O_2_ in water solution samples (Figure 9). The detection sensitivity was also compared to that received from the microplate-reader. The smartphone-based measurement was sensitive enough to detect 1% (*v*/*v*) ethanol, 0.02% chloroform (*v*/*v*), and 0.0006% (*v*/*v*) H_2_O_2_. The bacterial strain TV1061 immobilized on filter membrane disk demonstrated a dose-dependent detection pattern with 2.1, 3.1, and 7.6 times higher induction as compared to the control sample, when exposed to 1%, 2%, and 5% (*v*/*v*) ethanol, respectively (Figure 9A,B). The detection sensitivity that was received from the commercial microplate-reader was higher (22.6, 42.8, and 365.8-fold higher to 1%, 2%, and 5% (*v*/*v*) ethanol, respectively) than the one received in the smartphone-based setup. A similar pattern was also seen for the bacterial strain *grpE:lux* for the detection of ethanol. Specifically, the dose-dependent response patterns that were demonstrated were with sensitivity to 1%, 2%, and 5% (*v*/*v*) ethanol of 1.4, 1.7, and 2.0 times higher, respectively, as compared to the control. Meanwhile, a sensitivity of 3.6, 7.0, and 38.5 times higher was detected by the microplate-reader (Figure 9C,D). Likewise, the sensitivity of the bacterial strain DPD2794 (responsive to genotoxicants) to different concentrations (0.02%, 0.06%, and 0.15% (*v*/*v*)) of chloroform using the smartphone-based setup was 1.3, 1.4, and 1.4 times higher, respectively, as compared to the control; however, the detection sensitivity was lower than the one measured by the microplate-reader (2.1, 5.3, and 14.8 times higher induction factors, respectively) (Figure 9E,F). Similarly, the response patterns sensitivity of the bacterial strain *soxS::lux* to 0.0006%, 0.0012%, and 0.0018% (*v*/*v*) H_2_O_2_ were 1.9, 2.5, and 3.4 times higher than that of the control (Figure 9G,H). A comparable sensitivity of 2.4, 4.5, and 11.1 times higher was measured by the microplate-reader. 

Generally, the smartphone-based light signal detection was obtained with lower sensitivity when compared to the light signal detected by the microplate-reader. Unlike the Synergy HTX Multi-Mode microplate reader that is coupled with a highly sensitive but expensive PMT detector, the smartphone possesses only low-cost and less sensitive CMOS sensors [37,57]. This was also confirmed in the screening experiment of the *E. coli* bioreporter strains, where the smartphone could record detectable light signal only when the bioluminescent bacterial strains had a relatively higher basal signal intensity. Also, in the smartphone-based setup measurement process, the mean value of the signal intensity over 2000 s around the peak time (the time point with the maximum induction factor) was calculated as the final signal readout. This approach was chosen because the smartphone requires a much longer integration time and the stacking of multiple images in order to improve the signal-noise ratio [35,37]. Nevertheless, the immobilized bioreporter bacteria on the filter membrane disk integrated with the smartphone-based signal measurement setup could successfully discriminate between the different toxicants’ concentrations. The sensitivity that was achieved with the smartphone-based setup is sufficient and it also allows for rapid and qualitative analysis of environmental water toxicants. Furthermore, the use of an intervalometer application can also control the smartphone camera to enable real-time monitoring. Most importantly, the smartphone-based signal detection setup is more portable and low-cost, as compared to the bulky and expensive microplate-reader. This study findings demonstrate the proof-of-concept of immobilized WCBs with smartphone-based signal detection for environmental toxicants monitoring as well as for an additional variety of applications in quality monitoring of post-harvested agriculture products.

Toxicity monitoring usually show the integration of the bioreporter immobilized in either agar gel or calcium alginate beads, and with a commercial multimode plate reader or spectrophotometer [2,3,9]. These systems are often costly and bulky (20,000 US dollars). A cheaper and small-sized alternative (approximately 4000 US dollars) is the coupling with a CMOS photodetector [3]. The developed smartphone-based WCB system is even more cost-effective (approximately 300 US dollars) and portable; therefore, it is highly suitable to be used in low-resource regions. However, there are still challenges to be improved in the proposed system. Firstly, unlike agar or calcium alginate hydrogel that are usually used for the bioreporter immobilization, the filter membrane disk cannot maintain the moisture that is critical for the long-term storage of bacteria. Secondly, the smartphone-based measurement of the light signal is also restricted by its low sensitivity, and therefore it requires a certain threshold of light intensity. Another challenge is related to the water toxicity monitoring of real water samples that have a mixture of chemical toxicants or overconcentrated toxicants, which might have a matrix interference effect or requires pre-dilution, thereby lower the sensitivity, and introduce error or even affect the viability of the immobilized bacteria [58]. Thus, further testing is still needed to demonstrate the capability of the proposed WCB system for the monitoring of the combined effect of various toxicants [59]. 

## 4. Conclusions

In this study, a simple and low-cost method of the immobilization of bioluminescent bioreporter bacteria on filter membrane disks was examined. The tested immobilization strategy was further investigated as a WCB platform for the detection of environmental toxicants in water samples. The detection was compared to that in planktonic cells for ethanol, chloroform, and H_2_O_2_. The filter membrane disk maintained the functionality of the immobilized bioreporter bacteria for as many as three weeks, without compromising on its stability and its detection sensitivity level when stored at −20 °C. The WCBs were integrated with smartphone-based signal detection. To conclude, this study demonstrated a proof-of-concept for the construction of a portable, inexpensive monitoring device for a variety of toxicants in water samples. 

## Supplementary Materials

The Supplementary Materials of the Python script for the analysis of CMOS-generated data are available online at https://www.mdpi.com/1424-8220/20/19/5486/s1.
