# Supplementary material for: Smartphone-Based Whole-Cell Biosensor Platform Utilizing an Immobilization Approach on a Filter Membrane Disk for the Monitoring of Water Toxicants
Source: Sensors (Basel). 2020 Sep 25;20(19):5486. doi: 10.3390/s20195486 (PMC7582319; doi:10.3390/s20195486)
Supplement: Supplementary file 1 [file sensors-20-05486-s001.pdf]

Python script for the analysis of CMOS-generated data

(1) Data input and temperature reading extraction

```
import os

import pandas as pd

import numpy as np

import cv2

import matplotlib.pyplot as plt

# change of working directory to the place where the data file is.

os.chdir(r"C:\Users\CMOS ")

# read the data file using PandaFrame

df=pd.read_csv(r"CMOS-data.txt",header=None,names=range(13),sep='\s+')

# get the index of "temp"

t_index=[]

for i in range(len(df)):

    if df[0][i]=="Temp":

        t_index.append(i)

# get the temperature readout for each measurement

tem=[]

for j in range(len(t_index)):

    tem.append(df[2][t_index[j]])

     tem[j]=float(tem[j])

# cleaning the data by removing unwanted null value and transform the datatype

df.dropna(inplace=True)

df.drop(12,axis=1,inplace=True)

df.reset_index(drop=True,inplace=True)

df=df.astype(np.float)
```

(2) Data extraction for each channel

```
#get the data for each channel

a=df.to_numpy().flatten() # get the flattened numpy.array

b=np.split(a,len(a)/(12*12)) ## split the flattened array into equal number of sub-arrays (for each channel)

c1=np.array(b[0:len(b):4]) #all the reading from channel 1
```

```
c1=np.delete(c1,81,axis=0)
c2=np.array(b[1:len(b):4]) #all the reading from channel 2
c2=np.delete(c2,81,axis=0)
c3=np.array(b[2:len(b):4]) #all the reading from channel 3
c3=np.delete(c3,81,axis=0)
c4=np.array(b[3:len(b):4]) #all the reading from channel 4
c4=np.delete(c4,81,axis=0)
c1_b=np.zeros_like(c1) #Initialize a empety numpy array for channel_1_background signal
c2_b=np.zeros_like(c2) #Initialize a empety numpy array for channel_2_background signal
c3_b=np.zeros_like(c3)  #Initialize a empety numpy array for channel_3_background signal
c4_b=np.zeros_like(c4)  #Initialize a empety numpy array for channel_4_background signal
```

(3) Calculation of background signal reading according to the standard curve

#Calculate the background signal for each channel using the standard curve according to temperature

```
for i in range (len(tem)):
   c1_b[i]=c1_b[i]+(2**(0.082*tem[i]+6.6341))
   c2_b[i]=c2_b[i]+(2**(0.0747*tem[i]+7.07))
   c3_b[i]=c3_b[i]+(2**(0.0681*tem[i]+7.2837))
   c4_b[i]=c4_b[i]+(2**(0.0668*tem[i]+7.4708))
```

(4) Calibration of bacterial signal by subtraction of calculated background signal

#Calibrate signal by subtracting the background signal

```
c1=c1-c1_b
c2=c2-c2_b
c3=c3-c3_b
c4=c4-c4_b
```

# Normalize the image, to spread the pixel intensities from 0 to 255

```
c1_normalized = cv2.normalize(c1, None, 0, 255, cv2.NORM_MINMAX)
c2_normalized = cv2.normalize(c2, None, 0, 255, cv2.NORM_MINMAX)
c3_normalized = cv2.normalize(c3, None, 0, 255, cv2.NORM_MINMAX)
c4_normalized = cv2.normalize(c4, None, 0, 255, cv2.NORM_MINMAX)
```

(5) Computation of the induction factor

#Locate the number of images for the peak signal

induction_factor=[]

for i in range (len(c1)):

induction_factor.append((c4_normalized[i].mean()+c3_normalized[i].mean())/(c2_normalized [i].mean()+c1_normalized[i].mean()))

peak_signal_number=induction_factor.index(max(induction_factor))

Calculate the peak signal for each channel

peak_signal_c1=c1_normalized[peak_signal_number].mean()

peak_signal_c2=c2_normalized[peak_signal_number].mean()

peak_signal_c3=c3_normalized[peak_signal_number].mean()

peak_signal_c4=c4_normalized[peak_signal_number].mean()

Calculate the induction factor

induction_factor1=peak_signal_c3/peak_signal_c1

induction_factor2=peak_signal_c4/peak_signal_c2

(6) Reconstruction of the output images

To reconstruct the images

plt.figure(figsize=(8,8))

plt.subplot(221)

plt.imshow(c1_normalized[peak_signal_number].reshape((12,12)),vmin=50,vmax=220, cmap = 'gray', interpolation='nearest')

plt.title('Channel 1: control', y=1.02, fontsize=12)

plt.subplot(222)

plt.imshow(c2_normalized[peak_signal_number].reshape((12,12)),vmin=50,vmax=220, cmap = 'gray', interpolation='nearest')

plt.title('Channel 2: control', y=1.02, fontsize=12)

plt.subplot(223)

plt.imshow(c3_normalized[peak_signal_number].reshape((12,12)),vmin=50,vmax=220, cmap = 'gray', interpolation='nearest')

plt.title('Channel 3: 2% ethanol ', y=1.02, fontsize=12)

plt.subplot(224)

plt.imshow(c4_normalized[peak_signal_number].reshape((12,12)),vmin=50,vmax=220,

cmap = 'gray', interpolation='nearest')

```
plt.title('Channel 4: 2% ethanol ', y=1.02, fontsize=12)
plt.savefig("CMOS.tif", dpi=600)
```

Python script for stacking of smartphone-generated images

```
import os
import numpy as np
import matplotlib.pyplot as plt
import cv2

# read the images
folder=r"C:\Users\camera"
os.chdir(folder)
files=list([os.path.join(folder, f) for f in os.listdir(folder)])
new_files=[]
for i in range(len(files)):
    if (files[i].endswith(".jpg")):
        new_files.append(files[i])

# stacking the images by computing the average
average = cv2.imread(new_files[0]).astype(np.float)
for i in range(len(new_files)):
    average+=cv2.imread(new_files[i]).astype(np.float)
average=average/len(new_files)

# Normalize the image, to spread the pixel intensities from 0 to 255
# This will brighten the image without losing information
Stacked_image = cv2.normalize(average, None, 0, 255, cv2.NORM_MINMAX)
cv2.imwrite(stacked_image.png', Stacked_image)
plt.show()
```
